# A Case Report of Inguinal Hernia Containing the Appendix: Amyand's Hernia

**DOI:** 10.7759/cureus.68815

**Published:** 2024-09-06

**Authors:** Sanjay M Khaladkar, Urvashi Agarwal

**Affiliations:** 1 Radiodiagnosis, Dr. D. Y. Patil Medical College, Hospital and Research Centre, Dr. D. Y. Patil Vidyapeeth (Deemed to be University), Pune, IND

**Keywords:** amyand's hernia, appendix, computed tomography (ct) imaging, inguinal hernia, losanoff and basson's criteria

## Abstract

Numerous types of hernias have been reported in the literature. A unique and uncommon type of hernia known as an Amyand's hernia occurs when the inguinal sac contains the vermiform appendix. Due to its rarity, it is usually difficult to diagnose and often goes unreported. However, when it goes unnoticed and untreated, it can lead to complications such as strangulation and perforation. This is where medical imaging plays a pivotal role. This case study aims to provide an overview of Amyand's hernia while highlighting the vital role that imaging plays in diagnosing the condition, identifying any associated problems, characterizing the pathology, and classifying the hernia. This supports grading the severity and determining the appropriate course of management.

## Introduction

Any organ or portion of an organ that protrudes through an irregular gap in the wall or fascia that typically encloses it is referred to as a hernia [1]. Hernias can take many different forms, depending on their location and other variables. The most common type, inguinal hernias, usually involve bowel loops and/or omentum. An uncommon disorder known as an Amyand's hernia [1] occurs when the appendix is found in an inguinal hernia, with or without appendicitis. It is often asymptomatic and may not always be diagnosed. When inflamed, the appendix can cause complications and is frequently left untreated. Early preoperative diagnosis via computed tomography (CT) is crucial, as it helps surgeons manage patients more effectively and reduces morbidity and mortality rates. According to estimates, 0.4%-1% of occurrences of inguinal hernias result in Amyand's hernias [2]. Amyand's hernia-related appendicitis accounts for around 0.1% of all appendicitis cases [3].

## Case presentation

A 73-year-old male patient had been experiencing dysphagia, primarily with solid foods rather than liquids, for the past two months. He did not report any abdominal pain. A computed tomography (CT) scan of the thorax, abdomen, and pelvis revealed a well-defined soft tissue density lesion at the level of the cricopharynx, extending into the upper thoracic esophagus at the thoracic inlet. The liver, spleen, and adrenal glands were all normal, and the abdominal CT scan revealed no signs of metastases or enlargement of retroperitoneal lymph nodes.

**Figure 1:**
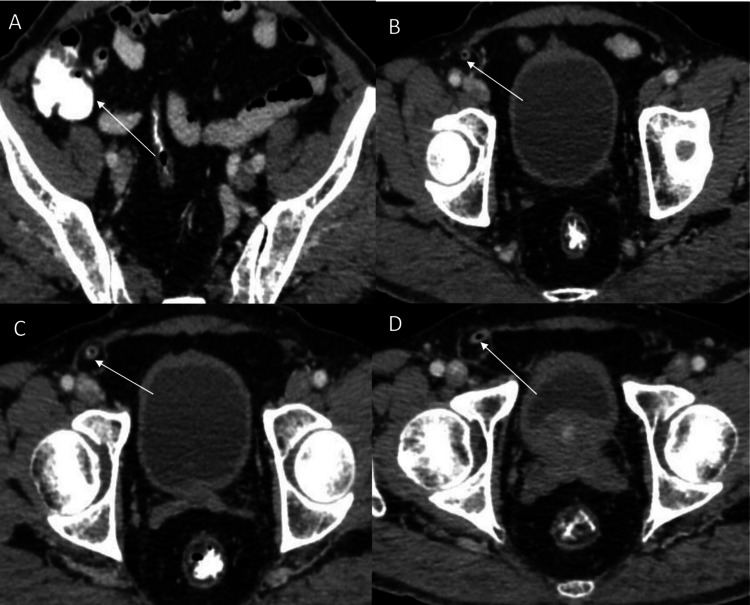
Axial CECT images showing cecum (A, white arrow), with the appendix arising from the cecum entering into the right inguinal canal with the presence of air within (B-D, white arrow) CECT: contrast-enhanced computed tomography

**Figure 2:**
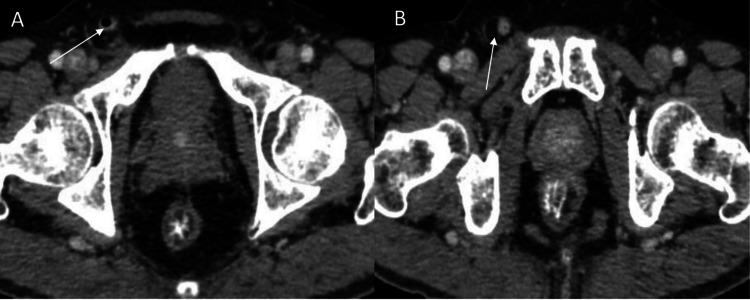
Axial CECT images showing the appendix arising from the cecum entering into the right inguinal canal (A and B, white arrow) CECT: contrast-enhanced computed tomography

**Figure 3:**
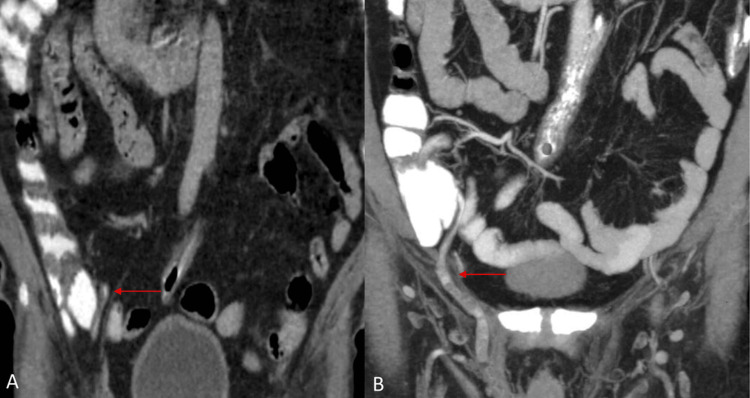
Reformatted coronal CT image showing the appendix arising from the cecum (A, red arrow) entering via the right inguinal hernia (B, red arrow) CT: computed tomography

An appendix was incidentally found within a right inguinal hernia. The hernia was partially filled with air and oral contrast, measuring approximately 5.5 mm in width (from the outer wall to the inner wall) and 8 cm in length (Figure 1). The peri-appendiceal fat planes appeared normal, and there was no indication of an appendicolith (Figure 2). There were no clinical or CT scan signs of appendicitis. The diagnosis was an indirect right-sided inguinal hernia, lateral to the inferior epigastric vessels, containing the omentum and appendix (Figure 3). Based on the criteria by Losanoff and Basson [4], this was identified as an inadvertently discovered type I Amyand's hernia.

Since the patient was asymptomatic and no complications were observed within the Amyand's hernia, the patient was asked for six monthly to yearly follow-ups.

## Discussion

The protrusion of the appendix into the inguinal sac is a characteristic of the atypical inguinal hernia known as Amyand's hernia [1]. It may present as asymptomatic, or if the appendix becomes inflamed, it can lead to incarceration, strangulation, or perforation. Clinical diagnosis is challenging and requires a high degree of suspicion, as it can resemble a strangulated hernia. Amyand's hernia is three times more common in the pediatric population due to the persistence of the processus vaginalis. Males are predominantly affected, with Amyand's hernias occurring in approximately 1% of pediatric patients [5,6]. Rarely, Amyand's hernias can be associated with conditions such as intestinal malrotation, situs inversus, or a movable cecum [1,4,5]. The hernia might result from a patent processus vaginalis, fibrous adhesions between the testicle and appendix, or congenital laxity of the right colon. While it can remain asymptomatic throughout a patient's life, it can sometimes cause severe swelling in the inguinal or inguinoscrotal area, mimicking an incarcerated hernia due to appendicitis. Necrotizing fasciitis is a rare but serious complication [7].

Losanoff and Basson's criteria are used for the management of Amyand's hernia [4]: type I, typical appendix; type II, acute appendicitis within the hernia sac; type III, peritonitis and acute appendicitis; and type IV, other abdominal diseases together with acute appendicitis [8,9].

Herniorrhaphy and the excision of the appendix are the recommended treatments for type I. Herniotomy and appendectomy through the hernia incision are recommended for type II. The usual procedure for types III and IV is a laparotomy followed by hernia surgery [8,9].

Amyand's hernias are often discovered incidentally during surgery. Advances in preoperative imaging, particularly CT scans, have made it easier to diagnose these hernias before surgery. Consequently, unexpected encounters with an inflamed appendix during the urgent treatment of strangulated inguinal hernias are becoming less common. Additionally, preoperative diagnostics facilitate laparoscopic techniques, which reduce the risk of surgical site infections [10].

In 1735, an 11-year-old male with an inguinal hernia and a severely inflamed appendix had the first successful appendectomy, conducted by Dr. Claudius Amyand [11]. The phrase "Amyand's hernia" celebrates an English surgeon who was born in France and described the situation when the appendix is inside the hernia sac, whether or not there is inflammation present. A case of an incarcerated hernia identified intraoperatively as an inflammatory appendix (type II Amyand's hernia) was documented by Shaban et al. [11]. They carried out an appendectomy and a polypropylene mesh hernia repair without using tension. Amyand's hernia cases were examined by Sözen et al. [12] in 21 instances. Right inguinal mass and right lower quadrant soreness were frequent observations, along with right inguinal mass and stomach pain as prevalent symptoms. Four individuals had neutrophilia and leukocytosis identified. Nine individuals had appendectomy with mesh hernioplasty and eight underwent appendectomy with hernioplasty.

Prosthetic material should be avoided in Amyand's hernia cases with inflammatory, suppurative, or perforated appendicitis because of the possibility of surgical site infection and fistula development from the appendicular stump. Because of its decreased recurrence rate, the Shouldice approach should be taken into consideration in addition to appendectomy [13].

## Conclusions

An Amyand's hernia is an inguinal hernia that contains the appendix. This relatively uncommon condition often goes unnoticed and is frequently discovered incidentally. However, misdiagnosis can lead to complications such as incarceration and strangulation. Computed tomography (CT) is not only the primary imaging modality but also the preferred diagnostic tool for identifying and classifying the type of hernia. Accurate classification with CT aids in making informed management decisions. Early and precise identification of this rare condition can significantly enhance the prognosis of affected patients.
